# Supplementary material for: Child Abuse and Neglect Awareness among Medical Students
Source: Children (Basel). 2022 Jun 14;9(6):885. doi: 10.3390/children9060885 (PMC9221984; doi:10.3390/children9060885)
Supplement: Supplementary file 1 [file children-09-00885-s001.zip › children-1735803-supplementary.pdf]

Table(s)

Table S1: Demographic details of the participants and awareness on child abuse and neglect.

Characteristic	N	%
Age (in years) Median	22.0	
(Interquartile range IQR)	(20.0,23.0)	
Mean±SD	21.75±1.6	
**Gender**		
Male	202	53.0
Female	179	47.0
**Academic year**		
Third	191	50.1
Sixth	190	49.9
**Are you aware about child abuse and neglect?**		
No	36	9.4
Yes	345	90.6
**Source of information on child abuse and neglect (Multiple response allowed)**		
Social media	311	37.6
Medical college	187	22.6
Relatives and friends	178	21.5
Mass media	152	18.4

Table S2: Participant's opinion on the importance and the level of prevalence of child abuse and neglect in saudi population.

Characteristic	N	%
**Do you think the topic on child abuse and neglect is important in medical field?**		
No	5	1.3
Yes	367	96.3
Uncertain	9	2.4
**Have you seen or encountered a case of child abuse and neglect during your medical study?**		
No	306	80.3
Yes	75	19.7
**Do you believe child abuse and neglect exists in Saudi's population?**		
No	6	1.6
Yes	368	96.6
Uncertain	7	1.8
**How common do you think child abuse and neglect is in Saudi's population?**		
Rare	28	7.3
Not common (neutral)	115	30.2
Common	174	45.7
Very common	64	16.8
**Do you think child abuse and neglect will affect his/her future?**		
No	2	0.5

Yes	372	97.6
Uncertain	7	1.8

Table S3: Participant's opinion on their responsibility in the issue and importance of courses or sessions on child abuse and neglect.

Characteristic	N	%
**As a future physician, generally at any discipline, do you think you have a role with child abuse and neglect?**		
No	11	2.9
Yes	321	84.3
Uncertain	49	12.9
**Do you have an interest to deal with child abuse and neglect?**		
No	63	16.5
Yes	217	57.0
Uncertain	101	26.5
**Have you taken an academic course(s) or session(s) about child abuse and neglect in your medical college?**		
No	271	71.1
Yes	110	28.9
**How good do you think those academic course(s) or session(s) about child abuse and neglect were?**		
Insufficient	19	17.3
Sufficient	20	18.2
Fair	56	50.9
Good	11	10.0

Excellent	4	3.6
**Do you like to receive more courses and sessions about child abuse and neglect?**		
No	33	8.7
Yes	268	70.3
Uncertain	80	21.0

Table S4: Participant's response on the legal ways child abuse and neglect is dealt and preventive measures.

**Characteristic**	**N**	**%**
**Who do you think deals with child abuse and neglect? (Multiple response allowed)**		
Social workers	347	25.5
Physicians	329	24.2
Police	306	22.5
Nurses	209	15.4
Administrators	169	12.4
**Do you think there is a legal system -legalization- to deal with child abuse and neglect in our kingdom?**		
No	57	15.0
Yes	324	85.0
**Who do you think abuse children the most (the abuser)?**		
Parents	219	57.5
Relatives (excluding parents)	51	13.4
Housemaids	83	21.8
Drivers (eg. school drivers or personal drivers)	12	3.1
Teacher	16	4.2

**Do you think that the child abuser has a psychological disorder?**		
Never	5	1.3
Rarely	11	2.9
Sometimes	119	31.2
Commonly	163	42.8
Always	69	18.1
Uncertain	14	3.7
**What do you think is the most effective preventive measure for child abuse and neglect?**		
Public education		
Medical staff education	102	26.8
School education	16	4.2
Legalization and law enforcement	29	7.6
	234	61.4

Table S5: Academic year response comparison.

CHARACTERESTICS	GENDER		P VALUE	ACADEMIC YEAR		P VALUE
	Males No: 202	FEMALES No: No: 179		3rd Y No: 191	6th Y No: 190	
	No. (%)	No. (%)		No. (%)	No. (%)	
**Are you aware about child abuse and neglect?**						
No	22 (10.9)	14 (7.8)	0.307	25 (13.1)	11 (5.8)	0.015
Yes	180 (89.1)	165 (92.2)		166 (86.9)	179 (94.2)	
**Do you think the topic on child abuse and neglect is important in medical field?**						
No	5 (2.5)	0 (0.0)	0.000*	4 (2.1)	1 (0.5)	0.019*
Yes	188 (93.1)	179 (100.0)		179 (93.7)	188 (98.9)	
Uncertain	9 (4.5)	0 (0.0)		8 (4.2)	1 (0.5)	
**Have you seen or encountered a case of child abuse and neglect during your medical study?**						
No	163 (80.7)	143 (79.9)	0.844	164 (85.9)	142 (74.7)	0.006
Yes	39 (19.3)	36 (20.1)		27 (14.1)	48 (25.3)	
**Do you believe child abuse and neglect exists in Saudi's population?**						
No	6 (3.0)	0 (0.0)	0.060*	4 (2.1)	2 (1.1)	0.386*
Yes	192 (95.0)	176 (98.3)		182 (95.3)	186 (97.9)	

Uncertain	4 (2.0)	3 (1.7)		5 (2.6)	2 (1.1)	
**How common do you think child abuse and neglect is in Saudi's population?**						
Rare	16 (7.9)	12 (6.7)	0.012	20 (10.5)	8 (4.2)	0.088
Not common (neutral)	74 (36.6)	41 (22.9)		60 (31.4)	55 (28.9)	
Common	86 (42.6)	88 (49.2)		80 (41.9)	94 (49.5)	
Very common	26 (12.9)	38 (21.2)		31 (16.2)	33 (17.4)	
**Do you think child abuse and neglect will affect his/her future?**						
No	2 (1.0)	0 (0.0)	0.631*	1 (0.5)	1 (0.5)	0.122
Yes	196 (97.0)	176 (98.3)		184 (96.3)	188 (98.9)	
Uncertain	4 (2.0)	3 (1.7)		6 (3.1)	1 (0.5)	
**As a future physician, generally at any discipline, do you think you have a role with child abuse and neglect?**						
No	10 (5.0)	1 (0.6)	0.000	8 (4.2)	3 (1.6)	0.000
Yes	154 (76.2)	167 (93.3)		143 (74.9)	178 (93.7)	
Uncertain	38 (18.8)	11 (6.1)		40 (20.9)	9 (4.7)	
**Do you have an interest to deal with child abuse and neglect?**						
No	38 (18.8)	25 (14.0)	0.286	27 (14.1)	36 (18.9)	0.277
Yes	108 (53.5)	109 (60.9)		116 (60.7)	101 (53.2)	

Uncertain	56 (27.7)	45 (25.1)		48 (25.1)	53 (27.9)	
**Have you taken an academic course(s) or session(s) about child abuse and neglect in your medical college?**						
No	138 (68.3)	133 (74.3)	0.198	169 (88.5)	102 (53.7)	0.000
Yes	64 (31.7)	46 (25.7)		22 (11.5)	88 (46.3)	
**How good do you think those academic course(s) or session(s) about child abuse and neglect were?**						1.000*
Insufficient	8 (12.5)	11 (23.9)	0.057*	4 (18.2)	15 (17.0)	
Sufficient	14 (21.9)	6 (13.0)		4 (18.2)	16 (18.2)	
Fair	35 (54.7)	21 (45.7)		11 (50.0)	45 (51.1)	
Good	7 (10.9)	4 (8.7)		2 (9.1)	9 (10.2)	
Excellent	0 (0.0)	4 (8.7)		1 (4.5)	3 (3.4)	
**Do you like to receive more courses and sessions about child abuse and neglect?**						
No	28 (13.9)	5 (2.8)	0.000	17 (8.9)	16 (8.4)	0.438
Yes	128 (63.4)	140 (78.2)		129 (67.5)	139 (73.2)	
Uncertain	46 (22.8)	34 (19.0)		45 (23.6)	35 (18.4)	

**Do you think there is a legal system -legalization- to deal with child abuse and neglect in our kingdom?**						
No	23 (11.4)	34 (19.0)	0.038	35 (18.3)	22 (11.6)	0.065
Yes	179 (88.6)	145 (81.0)		156 (81.7)	168 (88.4)	
**Who do you think abuse children the most (the abuser)?**						
Parents	101 (50.0)	118 (65.9)	0.001	106 (55.5)	113 (59.5)	0.001
Relatives (excluding parents)	35 (17.3)	16 (8.9)		34 (17.8)	17 (8.9)	
Housemaids	43 (21.3)	40 (22.3)		35 (18.3)	48 (25.3)	
Drivers (eg. school drivers or	9 (4.5)	3 (1.7)		10 (5.2)	2 (1.1)	
personal drivers)	14 (6.9)	2 (1.1)		6 (3.1)	10 (5.3)	
Teacher						
**Do you think that the child abuser has a psychological disorder?**						
Never	3 (1.5)	2 (1.1)	0.026*	5 (2.6)	0 (0.0)	0.000*
Rarely	7 (3.5)	4 (2.2)		8 (4.2)	3 (1.6)	
Sometimes	61 (30.2)	58 (32.4)		40 (20.9)	79 (41.6)	
Commonly	94 (46.5)	69 (38.5)		84 (44.0)	79 (41.6)	
Always	26 (12.9)	43 (24.0)		45 (23.6)	24 (12.6)	
Uncertain	11 (5.4	3 (1.7)		9 (4.7)	5 (2.6)	

**What do you think is the most effective preventive measure for child abuse and neglect?**						
Public education	63 (31.2)	39 (21.8)	0.047	50 (26.2)	52 (27.4)	0.919
Medical staff education	9 (4.5)	7 (3.9)		8 (4.2)	8 (4.2)	
School education	19 (9.4)	10 (5.6)		13 (6.8)	16 (8.4)	
Legalization and law enforcement	111 (55.0)	123 (68.7)		120 (62.8)	114 (60.0)	

Table S6: Comparison of the gender response in each academic year.

Characteristic	Academic year 3rd (N = 191)		P value	Academic year 6th (N = 190)		P value
	Male No. (%)	Female No. (%)		Male No. (%)	Female No. (%)	
**Are you aware about child abuse and neglect?**						
No	12 (12.1)	13 (14.1)	0.681	10 (9.7)	1 (0.5)	0.012
Yes	87 (87.9)	79 (85.9)		93 (90.3)	86 (98.9)	
**Do you think the topic on child abuse and neglect is important in medical field?**						
No	4 (4.0)	0 (0.0)	0.001*	1 (1.0)	0 (0.0)	1.000*
Yes	87 (87.9)	92 (100.0)		101 (98.1)	87 (100)	
Uncertain	8 (8.1)	0 (0.0)		1 (1.0)	0 (0.0)	

**Have you seen or encountered a case of child abuse and neglect during your medical study?**						
No	87 (87.9)	77 (83.7)	0.407	76 (73.8)	66 (75.9)	0.743
Yes	12 (12.1)	15 (16.3)		27 (26.2)	21 (24.1)	
**Do you believe child abuse and neglect exists in Saudi's population?**						
No	4 (4.0)	0 (0.0)	0.152*	2 (1.9)	0 (0.0)	0.251*
Yes	93 (93.9)	89 (96.7)		99 (96.1)	87 (100)	
Uncertain	2 (2.0)	3 (3.3)		2 (1.9)	0 (0.0)	
**How common do you think child abuse and neglect is in Saudi's population?**						
Rare	11 (11.1)	9 (9.8)	0.013	5 (4.9)	3 (3.4)	0.513*
Not common (neutral)	40 (40.4)	20 (21.7)		34 (33.0)	21 (24.1)	
Common	38 (38.4)	42 (45.7)		48 (46.6)	46 (52.9)	
Very common	10 (10.1)	21 (22.8)		16 (15.5)	17 (19.5)	

**Do you think child abuse and neglect will affect his/her future?**						
No	1 (1.0)	0 (0.0)	0.684*	1 (1.0)	0 (0.0)	0.707*
Yes	94 (94.9)	90 (97.8)		102 (99.0)	86 (98.9)	
Uncertain	4 (4.0)	2 (2.2)		0 (0.0)	1 (1.1)	
**As a future physician, generally at any discipline, do you think you have a role with child abuse and neglect?**						
No	7 (7.1)	1 (1.1)	0.000*	3 (2.9)	0 (0.0)	0.101*
Yes	61 (61.6)	82 (89.1)		93 (90.3)	85 (97.7)	
Uncertain	31 (31.3)	9 (9.8)		7 (6.8)	2 (2.3)	
**Do you have an interest to deal with child abuse and neglect?**						
No	18 (18.2)	9 (9.8)	0.227	20 (19.4)	16 (18.4)	0.698
Yes	56 (56.6)	60 (65.2)		52 (50.5)	49 (56.3)	
Uncertain	25 (25.3)	23 (25.0)		31 (30.1)	22 (25.3)	

**Have you taken an academic course(s) or session(s) about child abuse and neglect in your medical college?**	89 (89.9)	80 (87.0)	0.524	49 (47.6)	53 (60.9)	0.066
No	10 (10.1)	12 (13.0)		54 (52.4)	34 (39.1)	
Yes						
**How good do you think those academic course(s) or session(s) about child abuse and neglect were?**						
Insufficient	2 (20.0)	2 (16.7)	0.775*	6 (11.1)	9 (26.5)	0.043*
Sufficient	2 (20.0)	2 (16.7)		12 (22.2)	4 (11.8)	
Fair	6 (60.0)	5 (41.7)		29 (53.7)	16 (47.1)	
Good	0 (0.0)	2 (16.7)		7 (13.0)	2 (5.9)	
Excellent	0 (0.0)	1 (8.3)		0 (0.0)	3 (8.8)	

**Do you like to receive more courses and sessions about child abuse and neglect?**						
No	15 (15.2)	2 (2.2)	0.001	13 (12.6)	3 (3.4)	0.076
Yes	56 (56.6)	73 (79.3)		72 (69.9)	67 (77.0)	
Uncertain	28 (28.3)	17 (18.5)		18 (17.5)	17(19.5)	
**Do you think there is a legal system -legalization- to deal with child abuse and neglect in our kingdom?**						
No	14 (14.1)	21 (22.8)	0.121	9 (8.7)	13 (14.9)	0.183
Yes	85 (85.9)	71 (77.2)		94 (91.3)	74 (85.1)	
**Who do you think abuse children the most (the abuser)?**						
Parents	48 (48.5)	58 (63.0)	0.021*	53 (51.5)	60 (69.0)	0.028*
Relatives (excluding parents)	24 (24.2)	10 (10.9)		11 (10.7)	6 (6.9)	
Housemaids	15 (15.2)	20 (21.7)		28 (27.2)	20 (23.0)	
Drivers (eg. school or personal drivers)	7 (7.1)	3 (3.3)		2 (1.9)	0 (0.0)	
Teacher	5 (5.1)	1 (1.1)		9 (8.7)	1 (1.1)	

**Do you think that the child abuser has a psychological disorder?**						
Never	3 (3.0)	2 (2.2)	0.034*	0 (0.0)	0 (0.0)	0.577*
Rarely	5 (5.1)	3 (3.3)		2 (1.9)	1 (1.1)	
Sometimes	17 (17.2)	23 (25.0)		44 (42.7)	35 (40.2)	
Commonly	51 (51.5)	33 (35.9)		43 (41.7)	36 (41.4)	
Always	16 (16.2)	29 (31.5)		10 (9.7)	14 (16.1)	
Uncertain	7(7.1)	2 (2.2)		4 (3.9)	1 (1.1)	
**What do you think is the most effective preventive measure for child abuse and neglect?**						
Public education	27 (27.3)	23 (25.0)	0.097*	36 (35.0)	16 (18.4)	0.057*
Medical staff education	6 (6.1)	2 (2.2)		3 (2.9)	5 (5.7)	
School education	10 (10.1)	3 (3.3)		9 (8.7)	7 (8.0)	
Legalization and law enforcement	56 (56.6)	64 (69.6)		55 (53.4)	59 (67.8)	

* – Fisher's exact test
